# Serum lipoprotein (a) associates with a higher risk of reduced renal function: a prospective investigation

**DOI:** 10.1194/jlr.RA120000771

**Published:** 2020-10

**Authors:** Liping Xuan, Tiange Wang, Huajie Dai, Bin Wang, Jiali Xiang, Shuangyuan Wang, Hong Lin, Mian Li, Zhiyun Zhao, Jieli Lu, Yuhong Chen, Yu Xu, Weiqing Wang, Min Xu, Yufang Bi, Guang Ning

**Affiliations:** Department of Endocrine and Metabolic Diseases, Shanghai Institute of Endocrine and Metabolic Diseases, Ruijin Hospital, Shanghai Jiao Tong University School of Medicine, Shanghai, China, and Shanghai National Clinical Research Center for Metabolic Diseases, Key Laboratory for Endocrine and Metabolic Diseases of the National Health Commission of the PR China, Shanghai National Center for Translational Medicine, Ruijin Hospital, Shanghai Jiao Tong University School of Medicine, Shanghai, China

**Keywords:** hypertension, lipids, renal dysfunction, type 2 diabetes, epidemiology.

## Abstract

Lipoprotein (a) [Lp(a)] is a well-known risk factor for cardiovascular disease, but analysis on Lp(a) and renal dysfunction is scarce. We aimed to investigate prospectively the association of serum Lp(a) with the risk of reduced renal function, and further investigated whether diabetic or hypertensive status modified such association. Six thousand two hundred and fifty-seven Chinese adults aged ≤40 years and free of reduced renal function at baseline were included in the study. Reduced renal function was defined as estimated glomerular filtration rate <60 ml/min/1.73 m^2^. During a mean follow-up of 4.4 years, 158 participants developed reduced renal function. Each one-unit increase in log_10_-Lp(a) (milligrams per deciliter) was associated with a 1.99-fold (95% CI 1.15–3.43) increased risk of incident reduced renal function; the multivariable-adjusted odds ratio (OR) for the highest tertile of Lp(a) was 1.61 (95% CI 1.03–2.52) compared with the lowest tertile (*P* for trend = 0.03). The stratified analysis showed the association of serum Lp(a) and incident reduced renal function was more prominent in participants with prevalent diabetes [OR 4.04, 95% CI (1.42–11.54)] or hypertension [OR 2.18, 95% CI (1.22–3.89)]. A stronger association was observed in the group with diabetes and high Lp(a) (>25 mg/dl), indicating a combined effect of diabetes and high Lp(a) on the reduced renal function risk. An elevated Lp(a) level was independently associated with risk of incident reduced renal function, especially in diabetic or hypertensive patients.

Lipoprotein (a) [Lp(a)] consists of apo(a) bound covalently to apoB-100 of LDL-like particles (1, 2). Plasma Lp(a) mediates proatherogenic effects via LDL moiety, prothrombotic effects by the plasminogen-like apo(a), and proinflammatory responses via accumulation of oxidized phospholipids (3–6). Previous epidemiological and genetic studies have demonstrated that Lp(a) was associated with an increased risk of coronary heart disease, stroke, and vascular and nonvascular mortality (7–9).

Chronic kidney disease (CKD) has received increased attention as one of the leading public health problems, affecting 10–16% of the general adult populations in Asia, Europe, and the USA (10–13), and is associated with increased risk of mortality, cardiovascular diseases, and a progression to end-stage renal disease (10, 14). Decreased glomerular filtration rate (GFR) is the key kidney marker for definition of CKD (14).

An increase of Lp(a) concentrations was observed in the earliest stage of kidney impairment when GFR was not yet subnormal (15). Moreover, findings of several studies have also shown that increases in plasma Lp(a) levels occurred in patients with nonnephrotic kidney disease and those on hemodialysis (15–17). However, the effect of Lp(a) on the progression of CKD has not been evaluated yet. In fact, CKD frequently coexists with traditional cardiovascular risk factors, such as T2D and hypertension (18, 19). However, comprehensive analysis on the association of circulating Lp(a) levels with risk of reduced renal function in individuals with and without T2D or hypertension is scarce.

This prospective study aimed to prospectively assess the association of elevated serum Lp(a) concentrations with reduced renal function over a 4–5 year follow-up period in well-defined community study samples; in particular, we investigated whether diabetic or hypertensive status modifies such an association.

## MATERIALS AND METHODS

### Study population

The study participants were recruited from community residents of the Jiading district in Shanghai between March and August 2010. The design of this prospective cohort study has been described in detail earlier (20–22). Briefly, 10,375 of 10,569 registered permanent residents aged ≥40 years participated in the baseline examination for an investigation aimed to explore the effects of risk factors on T2D and related chronic diseases. Participants with missing data on serum creatinine (Scr) (n = 14) or serum Lp(a) (n = 9), or estimated GFR (eGFR) <60 ml/min/1.73 m^2^ (n = 309) at baseline were excluded and 10,043 participants were eligible for the prospective investigation. From August 2014 to May 2015, these 10,043 participants were invited to complete a follow-up examination. Two hundred and thirty participants died during the follow-up period, and 3,396 participants did not attend the follow-up onsite blood sampling and physical examination. Participants with missing data on measurements of Scr (n = 14) or serum Lp(a) (n = 146) at follow-up were further excluded, which subsequently left a total of 6,257 participants in the final analysis (supplemental Fig. S1).

The study abided by the principles of the Helsinki Declaration. The Institutional Review Board of Ruijin Hospital affiliated with Shanghai Jiao Tong University School of Medicine approved the study protocol. Written informed consent was obtained from each participant.

### Data collection and biochemical measurements

A standard questionnaire was used to collect the social demographic information, the history of chronic diseases and medications, and lifestyle factors. The current smoking or drinking status were defined as “yes” if the subject smoked cigarettes or consumed alcohol regularly in the past 6 months. Height and weight were measured to the nearest 0.1 kg and 0.1 cm separately with participants wearing lightweight clothes but without shoes. BMI was calculated as weight in kilograms divided by height squared in meters (kg/m^2^). Trained investigators measured systolic blood pressure (SBP) and diastolic blood pressure (DBP) in triplicate on the same day after a rest of at least 10 min by using an automated electronic device (OMRON model HEM-752 FUZZY; Omron Co., Dalian, China), and the average value of the three measurements was used for analysis.

At baseline and at the follow-up visit, all participants received standard 75 g oral glucose tolerances tests after an overnight fast of more than 10 h. Blood samples were obtained at 0 and 2 h during the test. Fasting plasma glucose (FPG) and 2 h post-loading plasma glucose (2 h PG) were measured by the glucose oxidase method using an autoanalyzer (Modular P800; Roche, Basel, Switzerland). Glycated hemoglobin (HbA1c) levels were determined by high-performance liquid chromatography (Bio-Rad, Hercules, CA).

Fasting serum total cholesterol (TC), triacylglycerol (TG), HDL-C, and LDL-C were measured by the chemiluminescence method with the auto-analyzer (Modular E170; Roche). The fasting Scr level was measured by using the picric acid method on an autoanalyzer (clinical chemistry diagnostic system C16000, Abbott Laboratories, Otawara-shi, Japan).

### Definitions of diabetes and hypertension

According to the American Diabetes Association 2010 Criteria, diabetes was defined as FPG ≥7.0 mmol/l (126 mg/dl), 2 h-oral glucose tolerance test PG ≥11.1 mmol/l (200 mg/dl), or HbA1c ≥6.5%, or previously diagnosed diabetes and receiving anti-diabetic therapy (23). The SBP ≥140 mmHg or DBP ≥90 mmHg, or those who were taking anti-hypertension medications were defined as hypertension.

### Measurement of Lp(a)

Serum Lp(a) levels were determined by murine monoclonal antibody (20-037, S0710-1; Jiemen BIO-TECH, Shanghai, China) by latex-enhanced immune transmission turbidimetry with a normal value of <30 mg/dl. For the laboratory test of serum Lp(a), the coefficient of variation within group was 8%, and the calibration of Lp(a) concentrations was validated by using a different antibody (Denka Seiken, Tokyo, Japan). More details on serum Lp(a) measurement are shown in our previous study (24).

### Assessment of incident reduced renal function

The 2009 Chronic Kidney Disease Epidemiology Collaboration (CKD-EPI) equation (25, 26) was used to calculate eGFR (expressed in milliliters per minute per 1.73 square meters), where Scr is serum creatinine concentration (in milligrams per deciliter) and age in years. The formulas were: *1*) If female: Scr ≤0.7 mg/dl, eGFR = 144 × (Scr /0.7)^−0.329^ × (0.993)^age^; Scr >0.7 mg/dl, eGFR = 144 × (Scr /0.7)^−1.209^ × (0.993)^age^. *2*) If male: Scr ≤0.9 mg/dl, eGFR = 141 × (Scr /0.9)^−0.411^ × (0.993)^age^; Scr >0.9 mg/dl, eGFR = 141 × (Scr /0.9)^−1.209^ × (0.993)^age^. Reduced renal function was defined as an eGFR of less than 60 ml/min per 1.73 m^2^ (11), with mildly decreased GFR defined as eGFR of 60–89 ml/min/1.73 m^2^. Participants without reduced renal function at baseline but defined as reduced renal function at the follow-up visit was categorized as incident reduced renal function.

### Statistical analysis

Participants were categorized into three groups according to tertiles of serum Lp(a) concentrations: tertile 1 with median of 7 mg/dl (0–11 mg/dl), tertile 2 with median of 18 mg/dl (12–25 mg/dl), and tertile 3 with median of 30 mg/dl (26–162 mg/dl). Data are presented as mean ± SD or if the distributions were skewed, median (25th–75th percentile) values for continuous variables and frequencies for categorical variables. The comparisons of baseline characteristics among groups were performed by one-way ANOVA for continuous variables, and χ^2^ test for categorical variables. *P* values for trend were calculated by using linear regression analyses and Cochran-Armitage trend test for continuous and categorical variables across the three groups, respectively. The skewed distribution variables, such as serum TG and Lp(a) data, were logarithmically transformed before statistical analysis.

Multivariable logistic regression analyses were used to assess the risk of incident reduced renal function in relation to serum Lp(a) concentrations in two models: model 1 was adjusted for sex, baseline age (years), and BMI (kilograms per square meter); model 2 was further adjusted for baseline FPG (millimoles per liter), SBP (millimeters of mercury), log_10_-TG (millimoles per liter), HDL-C (millimoles per liter), LDL-C (millimoles per liter), mildly decreased GFR (yes or no), current smoking and drinking status (yes or no), and use of antihypertensive drugs and antidiabetic drugs (yes or no). Odds ratio (OR) and the corresponding 95% CI were calculated in two models. In addition, we performed stratified analysis on the association between serum Lp(a) concentrations and incident reduced renal function according to baseline T2D and hypertension status.

For a more detailed exploration of the effect of combining Lp(a) and T2D, or hypertension status on the risk of reduced renal function, we categorized the participants into four groups according to low [≤25 mg/dl, equal to combination of Lp(a) tertile 1 and tertile 2] and high Lp(a) level [>25 mg/dl, equal to Lp(a) tertile 3], and T2D or hypertension status, respectively: *1*) non-T2D with low Lp(a), non-T2D with high Lp(a), T2D with low Lp(a), T2D with high Lp(a); *2*) nonhypertension with low Lp(a), nonhypertension with high Lp(a), hypertension with low Lp(a), hypertension with high Lp(a).

The generalized estimating equations were used to examine the regression coefficient (β) and 95% CIs for association of serum Lp(a) and eGFR. The two time-point (baseline and follow-up visit) measurements of serum Lp(a) were the independent variable, and the two time-point measurements of eGFR were the dependent variables. In this analysis, the multivariable adjustments included sex, age, BMI, FPG, SBP, LDL-C, HDL-C, log_10_-TG, smoking and drinking status, and use of antihypertensive drugs (not for the nonhypertension strata) and antidiabetic drugs (not for the non-T2D strata). Information on all covariates was updated at follow-up and modeled as repeated measures.

All analyses were conducted by using SAS version 9.4 (SAS Institute Inc, Cary, NC) and a two-sided *P* value of <0.05 was considered statistically significant.

## RESULTS

### Baseline characteristics of study population

The mean age of the 6,257 participants was 57.7 years (SD 8.6); 2,303 (36.8%) were men. Baseline characteristics of study participants according to tertiles of baseline serum Lp(a) concentrations are shown in **Table 1**. *P* for trend was calculated with each tertile of serum Lp(a) concentrations taken as a unit. The participants with the highest tertile of Lp(a) were less frequently men, smokers, alcohol drinkers, diabetics, hypertensives, and using antidiabetic drugs; had lower baseline BMI, SBP, DBP, FPG, TG, and eGFR but higher levels of LDL-C, HDL-C, TC, and higher prevalence of mildly decreased GFR compared with those with the lowest tertile of Lp(a) (all *P* for trend <0.05, Table 1).

**TABLE 1.** Baseline characteristics of the study participants stratified by tertiles of serum Lp(a)

Characteristics	Total Participants	Tertile 1	Tertile 2	Tertile 3	*P* for Trend
Lp(a), mg/dl	18 (0–162)	7 (0–11)	18 (12–25)	30 (26–162)	/
Number (%)	6,257	2,068 (33.1)	2,020 (32.3)	2,169 (34.6)	/
Age, years	57.8 ± 8.6	57.1 ± 8.7	57.9 ± 8.6	58.1 ± 8.5	0.001
Men, n (%)	2,303 (36.8)	865 (41.8)	736 (36.4)	702 (32.3)	<0.0001
BMI, kg/m^2^	25.2 ± 3.2	25.6 ± 3.3	25.1 ± 3.3	24.9 ± 3.16	<0.0001
SBP, mmHg	141.3 ± 19.7	142.6 ± 19.7	140.6 ± 19.4	140.5 ± 19.8	0.001
DBP, mmHg	83.1 ± 10.3	83.8 ± 10.3	82.7 ± 10.2	82.7 ± 10.3	0.0004
FPG, mmol/l	5.5 ± 1.5	5.7 ± 1.7	5.5 ± 1.5	5.4 ± 1.3	<0.0001
TG, mmol/l	1.4 (0.3–32.8)	1.5 (1.0–2.3)	1.4 (1.0–1.9)	1.3 (0.9–1.8)	<0.0001
LDL-C, mmol/l	3.2 ± 0.9	3.0 ± 0.8	3.2 ± 0.8	3.4 ± 0.9	<0.0001
HDL-C, mmol/l	1.3 ± 0.3	1.3 ± 0.3	1.3 ± 0.3	1.4 ± 0.3	<0.0001
TC, mmol/l	5.4 ± 1.0	5.2 ± 1.0	5.3 ± 1.0	5.5 ± 1.0	<0.0001
eGFR, ml/min/1.73 m^2^	90.9 ± 11.2	91.7 ± 11.3	90.7 ± 11.2	90.3 ± 11.0	<0.0001
Mildly decreased GFR, n (%)	2,693 (43.0)	831 (40.2)	882 (43.7)	980 (45.2)	0.001
T2D, n (%)	1,121 (17.9)	457 (22.1)	326 (16.1)	338 (15.6)	<0.0001
Hypertension, n (%)	3,752 (60.0)	1,316 (63.6)	1,168 (57.8)	1,268 (58.5)	0.001
Use of antidiabetic drugs, n (%)	465 (7.4)	184 (8.9)	143 (7.1)	138 (6.4)	0.002
Use of antihypertensive drugs, n (%)	1,739 (27.8)	591 (28.6)	538 (26.6)	610 (28.1)	0.75
Current smoker, n (%)	1,237 (20.0)	468 (22.8)	409 (20.4)	360 (16.8)	<0.0001
Current drinker, n (%)	630 (10.1)	252 (12.3)	202 (10.1)	176 (8.2)	<0.0001

Data are mean ± SD, median (interquartile range) for skewed variables, or n (proportion) for categorical variables. *P* for trend was calculated by using linear regression analyses and Cochran-Armitage trend test for continuous and categorical variables across the three groups, respectively./, no comparisons for Lp(a) levels or numbers (%) among groups.

### Associations of Lp(a) concentrations with risk of incident reduced renal function

During follow-up, 158 participants (2.5%) developed reduced renal function. The incidences of reduce renal function were 2.1, 2.4, and 3.0% from the lowest to the highest serum Lp(a) tertile, respectively. As shown in **Table 2**, each one-unit increase in log_10_-Lp(a) (milligrams per deciliter) was associated with a 1.81-fold (95% CI 1.08–3.01, *P* = 0.02) increased risk of incident reduced renal function after adjustment for age, sex, and BMI (model 1). After further adjustment for baseline FPG, SBP, log_10_-TG, HDL-C, LDL-C, mildly decreased GFR, smoking and drinking status, and use of antihypertensive drugs and antidiabetic drugs (model 2), the results did not appreciably change (OR = 1.99, 95% CI 1.15–3.43, *P* = 0.01). As compared with tertile 1, ORs for tertile 2 and tertile 3 of serum Lp(a) were 1.11 (95% CI 0.72–1.73) and 1.54 (95% CI 1.01–2.33) in model 1, respectively. In the fully adjusted model 2, the corresponding ORs and 95% CIs were 1.21 (0.76–1.92) and 1.61 (1.03–2.52) (all *P* for trend ≤0.03; Table 2).

**TABLE 2.** Association of serum Lp(a) concentrations with incident risk of reduced renal function

	Cases, n (%)	Model 1			Model 2		
		OR	95% CI	*P*	OR	95% CI	*P*
Continuous							
Log_10_-Lp(a)	158 (2.5)	1.81	1.08–3.01	0.02	1.99	1.15–3.43	0.01
Categorical							
Tertile 1	43 (2.1)	Reference			Reference		
Tertile 2	49 (2.4)	1.11	0.72–1.73	0.62	1.21	0.76–1.92	0.41
Tertile 3	66 (3.0)	1.54	1.01–2.33	0.04	1.61	1.03–2.52	0.03
*P* for trend		0.03			0.03		

Data are OR and 95% CI. Model 1 was adjusted for sex, baseline age, and BMI; model 2 was further adjusted for baseline FPG, SBP, log_10_-TG, HDL-C, LDL-C, mildly decreased GFR, smoking and drinking status, and use of antihypertensive drugs and antidiabetic drugs.

### Stratified analysis for associations of Lp(a) and incident reduced renal function by baseline diabetes and hypertension status

**Fig. 1.**
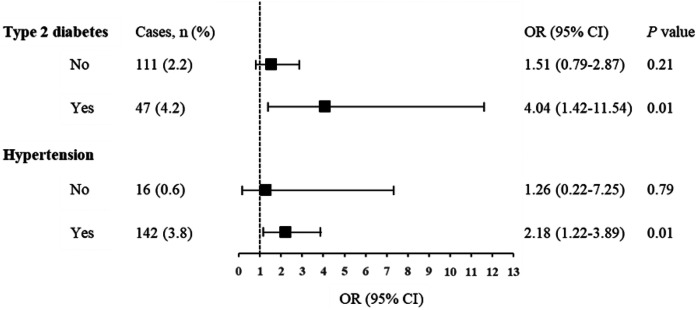
Association of baseline Lp(a) levels with incident reduced renal function stratified by diabetes and hypertension status. Data are ORs (95% CI) for of each one unit increase in log_10_-Lp(a). The model was adjusted for sex, baseline age, BMI, FPG, SBP, log_10_-TG, HDL-C, LDL-C, mildly decreased GFR, smoking and drinking status, and use of antihypertensive drugs (not for the nonhypertension strata) and antidiabetic drugs (not for the nondiabetes strata).

Furthermore, we conducted stratified analysis for associations of serum Lp(a) concentrations and incident reduced renal function according to baseline diabetes or hypertension status (**Fig. 1**). The incidence of reduced renal function in those with high Lp(a) was consistently higher than those with low Lp(a) within strata. The model was fully adjusted for sex, age, BMI, FPG, SBP, log_10_-TG, HDL-C, LDL-C, smoking and drinking status, and use of antihypertensive drugs (except for strata of nonhypertension) and antidiabetic drugs (except for strata of nondiabetes). Each one-unit increase in log_10_-Lp(a) concentrations was significantly associated with an increased risk of incident reduced renal function in the subgroup of T2D (OR = 4.04, 95% CI 1.42–11.54, *P* = 0.01) and hypertension (OR = 2.18, 95% CI 1.22–3.89, *P* = 0.01). There were no significant associations observed in the subgroup of non-T2D (OR = 1.51, 95% CI 0.79–2.87, *P* = 0.21) and nonhypertension (OR = 1.26, 95% CI 0.22–7.25, *P* = 0.79). No interactions were detected in the stratified analysis.

### Combined effect of Lp(a), T2D, and hypertension on reduced renal function

The incidence of reduced renal function according to combination of Lp(a) and T2D or hypertension status are summarized in **Table 3**. Compared with participants with low Lp(a) (≤25 mg/dl) and non-T2D, those with high Lp(a) (>25 mg/dl) and T2D had the highest ORs of 2.44 (95% CI 1.44–4.13, *P* = 0.001) in model 1 and 2.14 (95% CI 1.13–4.04, *P* = 0.02) in model 2 for reduced renal function. Similarly, the association between higher Lp(a) concentrations and the incidence of reduced renal function also achieved the most significant results in the group with high Lp(a) and hypertension with ORs of 4.71 (95% CI 2.19–10.15, *P* < 0.0001; model 1) and 3.09 (95% CI 1.31–7.29, *P* = 0.01; model 2; Table 3). In order to increase the number of participants with lower blood pressure, we used the upper quartile of blood pressure to recategorize the participants as the high blood pressure (SBP ≥154 mmHg or DBP ≥90 mmHg). There were 3,940 participants who were redefined as the lower blood pressure groups. The numbers of incident cases (n, %) of decreased renal function in the following groups: low blood pressure with low Lp(a) (36, 1.4%) or high Lp(a) (24, 1.7%), and high blood pressure with low Lp(a) (56, 3.7%) or high Lp(a) (41, 5.3%), are shown in supplemental Table S1. Similarly, a stronger association was observed in the group with high Lp(a) and high blood pressure with ORs of 2.84 (95% CI 1.75–4.62, *P* < 0.0001; model 1) and 2.43 (95% CI 1.46–4.02, *P* = 0.001; model 2). The results still indicated a combined effect of Lp(a) concentrations and high blood pressure on the reduced renal function risk.

**TABLE 3.** Combined effect of Lp(a) with T2D or hypertension on the risk of incident reduced renal function

	Cases, n (%)	Model 1			Model 2		
		OR	95% CI	*P*	OR	95% CI	*P*
Non-T2D							
Low Lp(a)	68 (2.1)	Reference			Reference		
High Lp(a)	43 (2.4)	1.21	0.80–1.82	0.36	1.17	0.76–1.79	0.46
T2D							
Low Lp(a)	24 (3.1)	1.04	0.63–1.73	0.86	0.87	0.45–1.70	0.69
High Lp(a)	23 (6.8)	2.44	1.44–4.13	0.001	2.14	1.13–4.04	0.02
Nonhypertension							
Low Lp(a)	8 (0.5)	Reference			Reference		
High Lp(a)	8 (0.9)	1.67	0.61–4.59	0.31	1.45	0.51–4.13	0.48
Hypertension							
Low Lp(a)	84 (3.4)	3.22	1.51–6.86	0.002	2.11	0.91–4.91	0.08
High Lp(a)	58 (4.6)	4.71	2.19–10.15	<0.0001	3.09	1.31–7.29	0.01

Data are OR and 95% CI. Participants were categorized into four groups by combining low and high Lp(a) with T2D or hypertension status, respectively. Low Lp(a) was defined as the combination of Lp(a) tertile 1 and tertile 2 (≤25 mg/dl), and high Lp(a) was otherwise defined as Lp(a) tertile 3 (>25 mg/dl). Model 1 adjusted for sex, baseline age, and BMI; model 2 further adjusted for baseline FPG, SBP, log_10_-TG, HDL-C, LDL-C, mildly decreased GFR, smoking and drinking status, and use of antihypertensive drugs and antidiabetic drugs.

### Associations of serum Lp(a) concentrations with eGFR

In addition, we assessed the associations of serum Lp(a) concentrations with eGFR (**Table 4**). After adjustment for the confounders, each one-unit increase in log_10_-Lp(a) and each 1-tertitle increase in Lp(a) were associated with a 1.04 ml/min/1.73 m^2^ (95% CI −1.67, −0.41, *P* = 0.001) and a 0.39 (95% CI −0.66, −0.12, *P* = 0.004) decrease in eGFR in total study participants. We further performed the stratified analysis according to baseline diabetes or hypertension status. The linear associations of log_10_-Lp(a) and eGFR were both found in nondiabetes (β = −0.77 ml/min/1.73 m^2^, 95% CI −1.47, −0.08, *P* = 0.03) and diabetes patients (β = −2.11, 95% CI −3.56, −0.66, *P* = 0.004). We also observed such an association among participants with prevalent hypertension (β = −1.15, 95% CI −1.95, −0.34, *P* = 0.01) but not in those without hypertension (β = −0.88, 95% CI −1.90, 0.12, *P* = 0.08) (Table 4).

**TABLE 4.** Association of Lp(a) concentrations with eGFR

	Each One-Unit Increase in log_10_-Lp(a)		Each One-Tertile Increase in Lp(a)	
	β (95% CI)	*P*	β (95% CI)	*P*
Total participants	−1.04 (−1.67, −0.41)	0.001	−0.39 (−0.66, −0.12)	0.004
T2D				
No	−0.77 (−1.47, −0.08)	0.03	−0.32 (−0.61, −0.02)	0.03
Yes	−2.11 (−3.56, −0.66)	0.004	−0.72 (−1.37, −0.06)	0.03
Hypertension				
No	−0.88 (−1.90, 0.12)	0.08	−0.32 (−0.74, 0.10)	0.14
Yes	−1.15 (−1.95, −0.34)	0.01	−0.46 (−0.81, −0.11)	0.01

The regression coefficient (β) and 95% CI were examined by linear regression models with generalized estimating equations, with the repeated measures of serum Lp(a) as the independent variable and the corresponding repeated measures of eGFR as the dependent variable. The adjustments included sex, age, BMI, FPG, SBP, log_10_-TG, HDL-C, LDL-C, smoking and drinking status, and use of antihypertensive drugs (not for the nonhypertension strata) and antidiabetic drugs (not for the non-T2D strata). Information on all covariates was updated at follow-up and modeled as repeated measures.

## DISCUSSION

In this prospective investigation in 6,257 community-dwelling Chinese adults, serum Lp(a) levels were significantly and independently associated with eGFR and risk of incident reduced renal function. Moreover, the association between Lp(a) and reduced renal function was more prominent among patients with diabetes or hypertension.

Previous studies suggested that an elevated Lp(a) level could be accompanied by renal dysfunction or increased albuminuria in diabetic or nondiabetic patients (15–17, 27, 28). Lp(a) concentrations increased significantly with decreasing GFR even in the earliest stages of renal impairment (15). A previous study of 217 patients with diabetes showed that patients with comorbidity of hypertension, coronary heart disease, microalbuminuria, or proteinuria had a statistically significant increased level of Lp(a); while the patients with hyper-Lp(a) (≥30 mg/dl) presented significantly increased levels of urea and TC (27). Moreover, several studies have demonstrated that Lp(a) was a significant prognostic factor for developing a new onset of CKD in diabetic patients (29–31). In a prospective study including 81 diabetic patients, the creatinine concentrations were significantly higher in patients with a Lp(a) level ≥30 mg/dl than those with a Lp(a) level <30 mg/dl after 1 year and 2 years of follow-up, respectively (29). Another two cohort studies (30, 31), including 862 patients and 581 patients with T2D, both demonstrated that Lp(a) level was an independent prognostic factor for the risk of CKD. In our present prospective investigation, we provided the evidence that an elevated Lp(a) level was an independent risk factor for the progression of reduced renal function in the general population and inversely associated with eGFR. This association was independent of hyperglycemia, hypertension, or lipid profile.

Emerging evidence has indicated that the prevalence of either hypertension or T2D always increases with decreased GFR (18, 19). Because both T2D and hypertension have a close relationship with CKD, we assumed that there might be a combined effect of Lp(a) with T2D and hypertension status on CKD; therefore, Lp(a) could further help to predict the risk of CKD in diabetic and hypertensive patients. In the current study, we not only analyzed the effect of Lp(a) in the general population but also assessed the combined effects of Lp(a) with T2D or hypertension status. Particularly, our results showed that high Lp(a) in individuals was more likely to have a stronger effect on reduced renal function when combined with diabetic status. Intriguingly, both the present study and our previous analysis (24) observed that T2D patients tended to have a lower Lp(a) level, indicating an inverse association between Lp(a) concentrations and T2D. Nevertheless, previous studies suggested that diabetes status did not attenuate the robust association between Lp(a) and cardiovascular risk (9, 29), and high glucose metabolism status plus elevated Lp(a) levels even had a higher risk for cardiovascular events (30). In the current analysis, we similarly found that the association between Lp(a) concentrations and reduced renal function risk was more prominent in patients with T2D or hypertension. It has also been demonstrated that Lp(a) was an independent risk factor for diabetic microvascular complications in patients with T2D (31–35), including diabetic nephropathy and retinopathy, which was in the line with our findings. Tu et al (35). investigated the association between Lp(a) concentration and diabetic retinopathy in patients with T2D and found that the patient group with the highest concentrations of both Lp(a) and HbA1c (≥7%) had a statistically significant OR for diabetic retinopathy compared with the patients with lower concentrations of both factors, indicating a combined effect of Lp(a) and HbA1c. Although the relatively fewer cases in the group with nonhypertension might not have enough power to indicate a combined effect of hypertension and Lp(a), the sensitive analysis still suggested a stronger association among participants with high Lp(a) and high blood pressure. Therefore, on considering the high prevalence of renal dysfunction in hypertensive patients (11, 18), paying more attention to the high Lp(a) levels in patients with hypertension was still recommended.

The mechanisms underlying the relationship between Lp(a) and renal dysfunction remain unclear. The arteriovenous differences in Lp(a) concentrations between arterial and renal veins and apo(a) fragments in urine were observed in previous studies, indicating that the kidney plays a role in the catabolism of Lp(a) (36, 37). Lp(a) quantitatively contains the atherogenic risk of LDL particles, which will oxidize after entry into the vessel wall, and then become highly immunogenic and proinflammatory oxidized LDL (38). Oxidized LDL is known to be toxic to vascular cells and may therefore lead to renal injury. Another main component of apo(a) also potentiates microvascular damage through additional mechanisms, including inflammation through its content of oxidized phospholipids (3). In addition to vascular injury, abnormalities in Lp(a) metabolism might be implicated in glomerular and tubulo-interstitial damage (39, 40). Further experimental studies are needed to clarify the causal relationship or pathogenic mechanism of Lp(a) abnormality with renal dysfunction.

Our study has the strengths of a relatively large sample size, a well-defined community setting, and a highly homogeneous population. To the best of our knowledge, our study was the first to assess the association between Lp(a) and the risk of renal dysfunction and the combined effect with T2D and hypertension. Several limitations of this study should be acknowledged when interpreting our findings. First, Lp(a) concentrations were not influenced very much by age, sex, and lifestyle factors but were under strict genetic control and highly associated with apo(a) isoforms (41). We did not measure apo(a) phenotypes or Lp(a) genotypes; therefore, the associations of apo(a) isoforms and Lp(a) genotypes with the progression of renal dysfunction remain to be defined. Second, the present analysis was based on a follow-up prospective design, which could not completely exclude the influence of the potential reverse causation. Previous studies observed an increase of Lp(a) in various kidney dysfunctions (15–17), even in the earliest stage of kidney impairment, indicating that renal dysfunction might elevate Lp(a). Elevated Lp(a) was also observed in participants with mildly decreased GFR at baseline in the present study. However, after adjusting for baseline mildly decreased GFR status, the positive association between Lp(a) concentrations and the risk of reduced renal function was still significant. Nevertheless, a prospective investigation with longtime follow-up in a larger sample size cohort or a Mendelian randomization study that may help to assess the causal link are needed. Third, we used the 2009 CKD-EPI equation to estimate the GFR, rather than the technetium 99m diethylene-triaminepentaacetic acid (99mTc-DTPA) renal dynamic imaging method. However, the accuracy of the CKD-EPI equation has already been validated and confirmed in previous studies (42, 43). Finally, our study was limited to the Chinese middle-aged and elderly population. It was reported that the lower Lp(a) levels were much lower in Chinese than in other ethnicity groups (44), so the results might not be generalizable to younger people and other ethnicities.

In conclusion, serum Lp(a) was an independent risk factor of incident reduced renal function in middle-aged and elderly Chinese. Moreover, the association between Lp(a) and reduced renal function was more prominent among patients with diabetes or hypertension, highlighting the importance of measurements of Lp(a) and treatment strategies toward clinical practice and management of Lp(a)-hyperlipoproteinemia.

### Data availability

Data are available from the authors on request.

## Supplementary Material

Supplemental Data
