# Supplementary material for: Serum lipoprotein (a) associates with a higher risk of reduced renal function: a prospective investigation
Source: J Lipid Res. 2020 Oct;61(10):1320–7. doi: 10.1194/jlr.RA120000771 (PMC7529054; doi:10.1194/jlr.RA120000771)
Supplement: Supplemental Data [file supp_61_10_1320__index.html]

Serum lipoprotein (a) associates with a higher risk of reduced renal function: a prospective investigation — Supplemental Data 

# Serum lipoprotein (a) associates with a higher risk of reduced renal function: a prospective investigation

## Supplemental Data

- Supplementary Data - Supplementary Figure 1. Flow chart of study participants. eGFR: estimated glomerular filtration rate. Supplementary Figure 2. Frequency distribution of serum Lp (a) concentrations by reduced renal function status. Supplementary Table 1. Combined effect of Lp (a) with high blood pressure on the risk of incident reduced renal function.
