# Supplementary material for: Serum lipoprotein (a) associates with a higher risk of reduced renal function: a prospective investigation
Source: J Lipid Res. 2020 Oct;61(10):1320–7. doi: 10.1194/jlr.RA120000771 (PMC7529054; doi:10.1194/jlr.RA120000771)
Supplement: Supplemental Data [file supp_RA120000771_159600_1_supp_552843_qcc53m.docx]

**Figure Legends.**

**Supplementary Figure 1.** **Flow chart of study participants. eGFR: estimated glomerular filtration rate.**

**Supplementary Figure 2.** **Frequency distribution of serum Lp (a) concentrations by reduced renal function status. A: Frequency distribution of Lp (a) in all participants; B: Frequency distribution of Lp (a) in reduced renal function; C: Frequency distribution of Lp (a) in non-reduced renal function.**

**Supplementary Figure 1.**

**
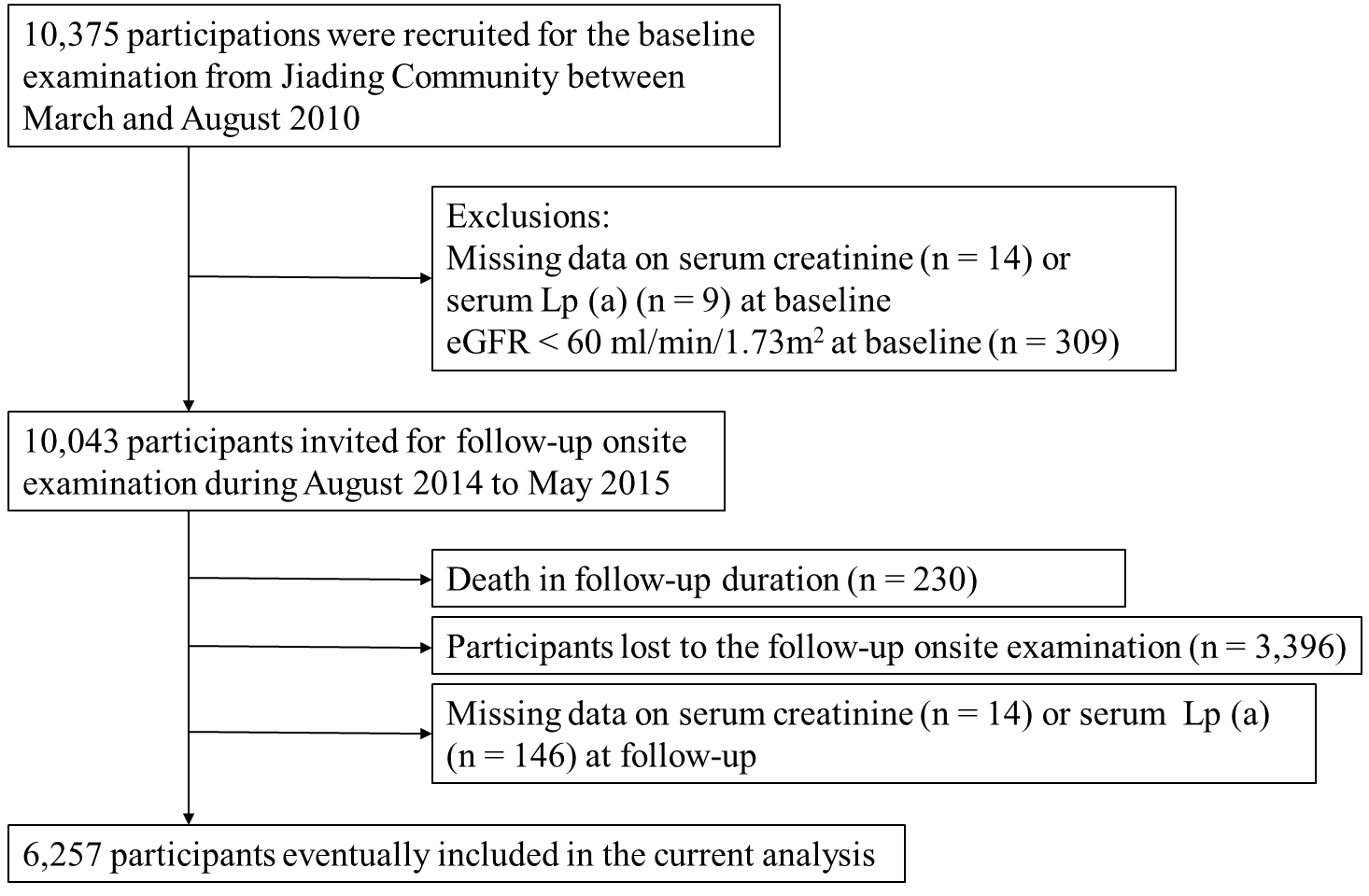
**

**Supplementary Figure 2.**


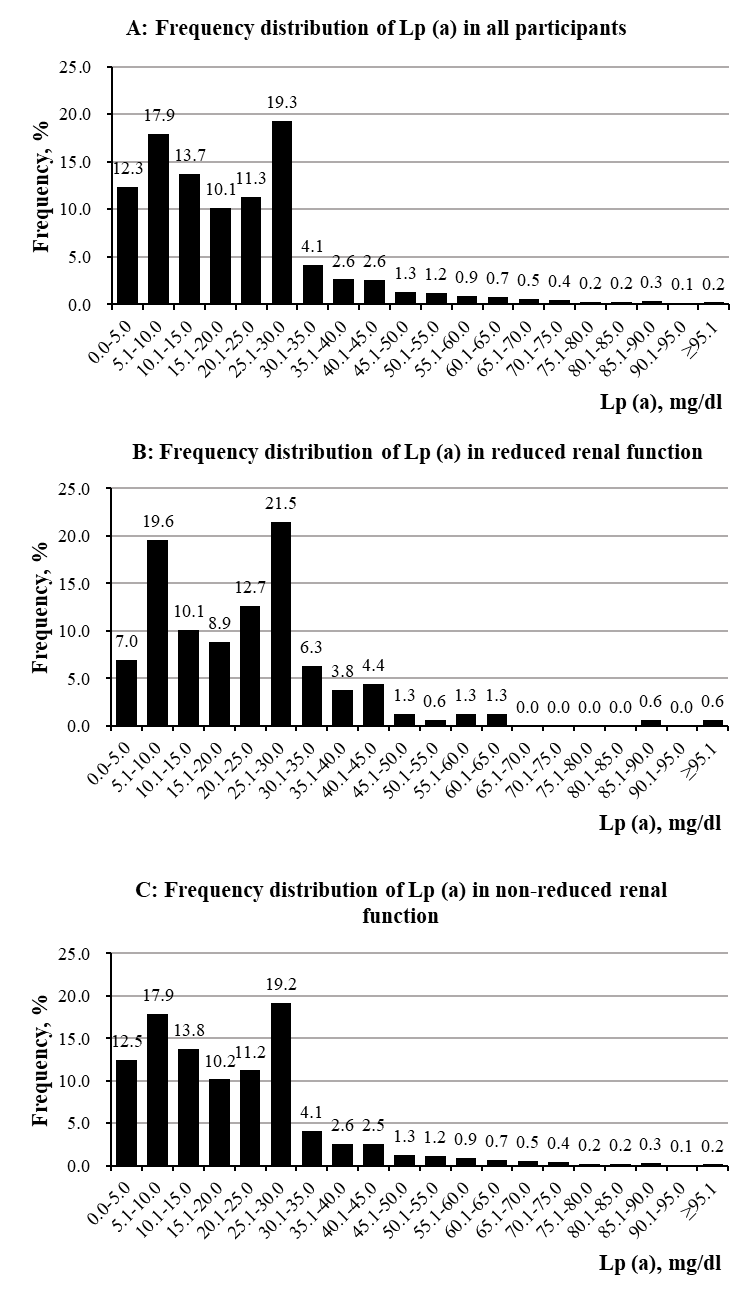


**Supplementary Table 1. Combined effect of Lp (a) with high blood pressure on**

|  | Cases, n (%) |  | Model 1 | | |  | Model 2 | | |
| --- | --- | --- | --- | --- | --- | --- | --- | --- | --- |
|  |  |  | OR | 95% CI | *P* |  | OR | 95% CI | *P* |
| Low blood pressure |  |  |  |  |  |  |  |  |  |
| Low Lp (a) | 36 (1.4) |  | Ref. |  |  |  | Ref. |  |  |
| High Lp (a) | 24 (1.7) |  | 1.29 | 0.75-2.22 | 0.36 |  | 1.28 | 0.74-2.22 | 0.37 |
| High blood pressure |  |  |  |  |  |  |  |  |  |
| Low Lp (a) | 56 (3.7) |  | 1.83 | 1.16-2.88 | 0.01 |  | 1.55 | 0.98-2.47 | 0.06 |
| High Lp (a) | 41 (5.3) |  | 2.84 | 1.75-4.62 | <0.0001 |  | 2.43 | 1.46-4.02 | 0.001 |

**the risk of incident reduced renal function.**

Data are odds ratio (OR) and 95% confidence interval (CI). Participants were categorized into two groups by combining low and high Lp (a) with blood pressure. The upper quartile of blood pressure was used to define the high blood pressure with SBP ≥154 mmHg or DBP ≥90 mmHg, and the low blood pressure with SBP <154 mmHg and DBP <90 mmHg. Low Lp (a) was defined as the combination of Lp(a) tertile 1 and tertile 2 (≤25 mg/dl), and high Lp (a) was otherwise defined as Lp (a) tertile 3 (>25 mg/dl). Model 1 adjusted for sex, baseline age, and BMI; Model 2 further adjusted for baseline FPG, log_10_-TG, HDL-C, LDL-C, mildly decreased GFR, smoking and drinking status, and use of antihypertensive drugs and antidiabetic drugs.
